# 755‐nm Picosecond Laser Versus Intense Pulsed Light for Diffuse Lip Hyperpigmentation: A Prospective, Randomized, Controlled, Evaluator‐Blinded Trial

**DOI:** 10.1111/jocd.71295

**Published:** 2026-11-13

**Authors:** Hongfei Ouyang, Jiahui Han, Lili Chen, Xuejiao Song, Yong Cui, Ruixing Yu

**Affiliations:** ^1^ China‐Japan Friendship Hospital (Institute of Clinical Medical Sciences) Chinese Academy of Medical Sciences & Peking Union Medical College Beijing China; ^2^ Department of Dermatology Peking University First Hospital Beijing China; ^3^ Department of Dermatology China‐Japan Friendship Hospital Beijing China; ^4^ China‐Japan Friendship Institute of Clinical Medical Sciences Beijing China; ^5^ Big Data Center, Department of Information China‐Japan Friendship Hospital Beijing China; ^6^ NHC Key Laboratory of Clinical Big Data Standardization & Integration Beijing China

**Keywords:** 755‐nm picosecond laser, efficacy, intense pulsed light, lip hyperpigmentation, safety

## Abstract

**Background:**

Lip hyperpigmentation is an aesthetic concern. Evidence supporting light‐based treatments for diffuse lip hyperpigmentation is limited. This study evaluated the efficacy and safety of 755‐nm picosecond laser versus intense pulsed light (IPL) for the treatment of diffuse lip hyperpigmentation.

**Methods:**

In this 12‐week, prospective, randomized, controlled, evaluator‐blinded trial, 50 patients were assigned 1:1 to five sessions of either a 755‐nm picosecond laser or IPL at 2‐week intervals. The primary endpoint was the Physician's Global Assessment (PGA) score at Week 12. Secondary endpoints included objective colorimetric parameters (CIE L*, a*, b*), Global Aesthetic Improvement Scale (GAIS), Dermatology Life Quality Index (DLQI), and pain Numerical Rating Scale (NRS). Treatment‐related adverse events were also recorded.

**Results:**

Forty‐four patients completed the study (IPL, *n* = 20; picosecond laser, *n* = 24). Among completers, the median PGA score at Week 12 was higher in the picosecond laser group than in the IPL group (2.5 [IQR, 2–3] vs. 1.5 [IQR, 1–2.375]; *p* = 0.007; Hodges–Lehmann estimate, 0.5 PGA points; 95% CI, 0–1.0), and more patients achieved at least moderate improvement (79.2% vs. 45.0%). In sensitivity analyses, statistical significance depended on assumptions about missing PGA outcomes. Objective colorimetric changes did not differ significantly between groups (all *p* > 0.05). All completers reported improvement on the GAIS, and DLQI scores decreased in both groups. Pain NRS scores were higher in the picosecond laser group (*p* < 0.01). Transient scaling occurred in 2 ps‐laser recipients; no adverse events were reported with IPL.

**Conclusion:**

Both the 755‐nm picosecond laser and IPL showed improvement in diffuse lip hyperpigmentation, with favorable safety profiles. The picosecond laser showed greater physician‐assessed improvement among completers, whereas IPL was better tolerated. These findings may help guide individualized treatment selection.

**Trial Registration:**

Chinese Clinical Trial Registry, ChiCTR2600116083

## 1 Introduction

Lip hyperpigmentation is a common aesthetic concern characterized by localized or diffuse darkening of the lips. It predominantly affects individuals with higher Fitzpatrick skin types, including Asian, Middle Eastern, African American, and Hispanic populations [1, 2]. Its etiology is multifactorial and includes physiological pigmentation, post‐inflammatory hyperpigmentation (PIH), smoking, ultraviolet exposure, drug‐induced pigmentation (e.g., antimalarials and minocycline), and pigmentation associated with systemic syndromes [3].

Physiological lip hyperpigmentation (PLH) is one of the most common forms. It is primarily caused by increased melanocyte activity and melanin deposition in the epidermis, while the number of melanocytes remains normal [4]. Clinically, PLH presents as diffuse, symmetric light to dark brown macules on the lips and may also involve the gingiva, buccal mucosa, and tongue in some cases [1, 5]. Lip darkening is often perceived as aesthetically undesirable and rarely resolves spontaneously, thereby negatively affecting appearance and psychological well‐being [3, 6].

Current management strategies for lip hyperpigmentation include removal of triggering factors (e.g., smoking cessation, photoprotection, and adjustment of suspected medications), cosmetic camouflage, and energy‐based therapies [3]. Lipstick and lip tattooing are commonly used camouflage methods. However, their effects are temporary, and long‐term or frequent use may increase the risk of adverse events such as contact dermatitis, granulomatous reactions, infection, and even malignancy [7].

Energy‐based devices are widely used for aesthetic and depigmentation treatments and have shown efficacy in various pigmentary disorders, including melasma, PIH, and periorbital hyperpigmentation [8, 9, 10]. However, clinical evidence for their use on the lips remains limited. Notably, the lips differ substantially from facial skin in anatomical structure and barrier function. The vermilion has a thinner stratum corneum, lower melanocyte density, and abundant vasculature, resulting in a relatively weak barrier and increased sensitivity to external stimuli and pain [11]. These characteristics suggest that treatment strategies for the lips may differ from those used for other facial regions.

Laser therapy has been reported to be effective in treating lip hyperpigmentation, particularly for localized lesions such as labial lentigines, Peutz–Jeghers syndrome, and Laugier–Hunziker syndrome [12, 13]. Commonly used lasers include Q‐switched Nd:YAG (532 and 1064 nm) and Q‐switched alexandrite (755 nm) lasers [12, 13, 14]. These devices target melanin through subcellular selective photothermolysis with nanosecond pulse durations [15]. Nevertheless, treatment is often associated with adverse effects, including significant pain, erythema, swelling, mild bleeding, and crusting [12].

At present, there is no established consensus or guidelines for the standardized treatment of diffuse lip hyperpigmentation. The 755‐nm picosecond laser delivers ultrashort pulses with high peak power, disrupting pigment particles predominantly through photomechanical effects while limiting thermal diffusion [16]. In contrast, intense pulsed light (IPL) is a noncoherent, broad‐spectrum light source that uses wavelength‐selective filters to target melanin through selective photothermolysis and is widely used for superficial pigmentary disorders and photoaging [17]. However, comparative evidence regarding these two nonablative modalities in diffuse lip hyperpigmentation remains lacking. Therefore, this prospective, randomized controlled trial aimed to compare the efficacy and safety of a 755‐nm picosecond laser and IPL in patients with diffuse lip hyperpigmentation.

## 2 Materials and Methods

### 2.1 Study Design

This study was a 12‐week prospective, randomized, controlled, evaluator‐blinded trial. No formal sample size calculation was performed because of the exploratory nature of the study. Patients with diffuse lip hyperpigmentation were randomized 1:1 to receive IPL or 755‐nm picosecond laser treatment. Randomization was performed by an independent investigator using a computer‐generated random sequence in Microsoft Excel. Participants and treating physicians were not blinded, whereas outcome assessors were blinded to treatment allocation. The study protocol was approved by an institutional ethics committee and registered in a publicly accessible clinical trial registry. Written informed consent was obtained from all participants before enrollment. This study was designed and reported in accordance with the Consolidated Standards of Reporting Trials (CONSORT) statement and conducted in accordance with the Declaration of Helsinki [18].

### 2.2 Inclusion and Exclusion Criteria

Participants aged 18–60 years, regardless of sex, with clinically diagnosed benign diffuse lip hyperpigmentation presenting as uniform light to dark brown discoloration were eligible for inclusion.

Exclusion criteria were pregnancy or lactation; smoking or drug‐induced pigmentation (e.g., associated with antimalarial agents or minocycline); active lip lesions (e.g., herpes, inflammation, or tumors); lip treatments within the previous 3 months, including fillers, chemical peels, or energy‐based procedures; lip tattooing; focal pigmentary lesions or syndromic pigmentation (e.g., Peutz–Jeghers syndrome or Laugier–Hunziker syndrome); endocrine or metabolic disorders (e.g., Addison's disease, Cushing's syndrome, or hyperthyroidism); photosensitivity; a history of keloid formation; a history of skin cancer or radiotherapy; and inability to comply with strict photoprotection.

### 2.3 Treatment Protocol and Follow‐Up

Patients in the IPL group were treated with an intense pulsed light system (M22; Lumenis, USA) on the upper and lower lips. Treatment parameters were as follows: a 560‐nm filter, fluence 10–12 J/cm^2^, three sub‐pulses, pulse duration 4 ms, delay 40 ms, and one pass. A thin layer of ultrasound gel was applied before irradiation. Patients in the picosecond laser group were treated with a 755‐nm alexandrite picosecond laser (PicoSure; Cynosure, USA) on the upper and lower lips. Treatment parameters were as follows: wavelength 755 nm, spot size 8 mm, fluence 0.4 J/cm^2^, and frequency 10 Hz. In each treatment session, one pass was performed with a standard handpiece and one with a FOCUS diffractive lens array, with approximately 75 ± 25 pulses delivered in each pass. For both treatments, the clinical endpoint was mild to moderate erythema of the lip skin. Post‐treatment care consisted of a 15‐min ice compress, followed by the application of petrolatum‐based lip balm and avoidance of sun exposure.

Patients were scheduled to receive five treatment sessions at 2‐week intervals. Assessments were performed at baseline, 2 weeks after each treatment session, and 4 weeks after the final session, for a total study duration of 12 weeks. Standardized facial photographs were obtained using the VISIA imaging system (Canfield Scientific, USA) under standard white light and cross‐polarized light. Before imaging, patients cleansed their face and lips with a facial cleanser and rested for 15 min.

### 2.4 Clinical Evaluation

At baseline, the severity of lip hyperpigmentation was graded according to the classification proposed by Altalhab et al. [4]: Grade 1, diffuse hyperpigmentation of the upper lip and the border of the lower lip; Grade 2, involvement of the upper lip and most of the lower lip with relative sparing of the central portion; and Grade 3, near‐complete involvement of both lips.

The primary endpoint was the Physician's Global Assessment (PGA) score at Week 12. PGA was independently assessed by two dermatologists blinded to treatment allocation but aware of the image time points (baseline and Week 12). Improvement was graded on a quartile scale: 0 = no improvement; 1 = mild improvement (≤ 25%); 2 = moderate improvement (26%–50%); 3 = good improvement (51%–75%); and 4 = excellent improvement (> 75%). The final PGA score was calculated as the mean of the two assessors' ratings.

Secondary objective outcomes were changes from baseline to Week 12 in colorimetric parameters, including lightness (L*), red–green axis (a*), and yellow–blue axis (b*). L* reflects skin lightness, a* reflects erythema, and b* reflects pigmentation [19]. Cross‐polarized VISIA images were converted from RGB to the 1976 CIELAB color space and analyzed using ImageJ software (National Institutes of Health, USA; version 1.54 g). Measurements were obtained from standardized regions of interest (ROIs), and mean values were calculated separately for the upper and lower lips; detailed ROI placement is shown in Figure S1.

Patient‐reported outcomes included the Global Aesthetic Improvement Scale (GAIS), Dermatology Life Quality Index (DLQI), and pain Numerical Rating Scale (NRS). GAIS was categorized as worse, no change, improved, much improved, and very much improved. DLQI was assessed at baseline and Week 12. For the first treatment session, pain NRS scores (0–10) were recorded immediately after treatment and after 15 min of post‐treatment cooling [20].

Safety was assessed by recording treatment‐related adverse events throughout the study, including erythema, scaling, edema, blistering, crusting, hypopigmentation, and hyperpigmentation.

### 2.5 Statistical Analysis

Statistical analyses were performed using SPSS (version 31.0), GraphPad Prism (version 10) and Python (version 3.12). *p* < 0.05 was considered statistically significant. Continuous variables were presented as mean ± standard deviation (SD) or median (interquartile range, IQR), and categorical variables as counts (percentages). Analyses of PGA, colorimetric parameters, GAIS, and DLQI were based on the 44 participants who completed the study. Pain NRS analyses included all 50 randomized participants with first‐session pain assessments. CIE L*, a*, and b* values were analyzed using paired *t*‐tests for within‐group comparisons, and percentage changes from baseline were compared between groups using Welch's *t*‐test. Inter‐rater reliability of PGA scores was assessed using a two‐way random‐effects, absolute‐agreement intraclass correlation coefficient (ICC) for the mean of two ratings [ICC(2,2)], with a 95% confidence interval (CI). PGA scores, DLQI scores, and NRS scores were analyzed using nonparametric tests. Within‐group comparisons were performed using the Wilcoxon signed‐rank test, and between‐group comparisons using the Mann–Whitney U test. Between‐group differences (picosecond laser minus IPL) were estimated using the Hodges–Lehmann method with nominal 95% CIs. Risk differences and risk ratios were reported with Newcombe–Wilson and Katz 95% CIs, respectively. GAIS distributions were compared using Fisher's exact test. Safety was assessed in all participants who received at least one treatment session.

Sensitivity analyses included all 50 participants in their randomized groups, using alternative assumptions about missing Week‐12 PGA scores and response status (PGA ≥ 2). Comparisons used Mann–Whitney *U* and Fisher's exact tests, respectively.

## 3 Results

### 3.1 Demographic and Clinical Data

A total of 50 patients were randomized (IPL, *n* = 25; picosecond laser, *n* = 25), and 44 completed the study (IPL, *n* = 20; picosecond laser, *n* = 24) (Figure 1). Demographic and clinical characteristics of all randomized participants are summarized in Table 1.

**FIGURE 1:**
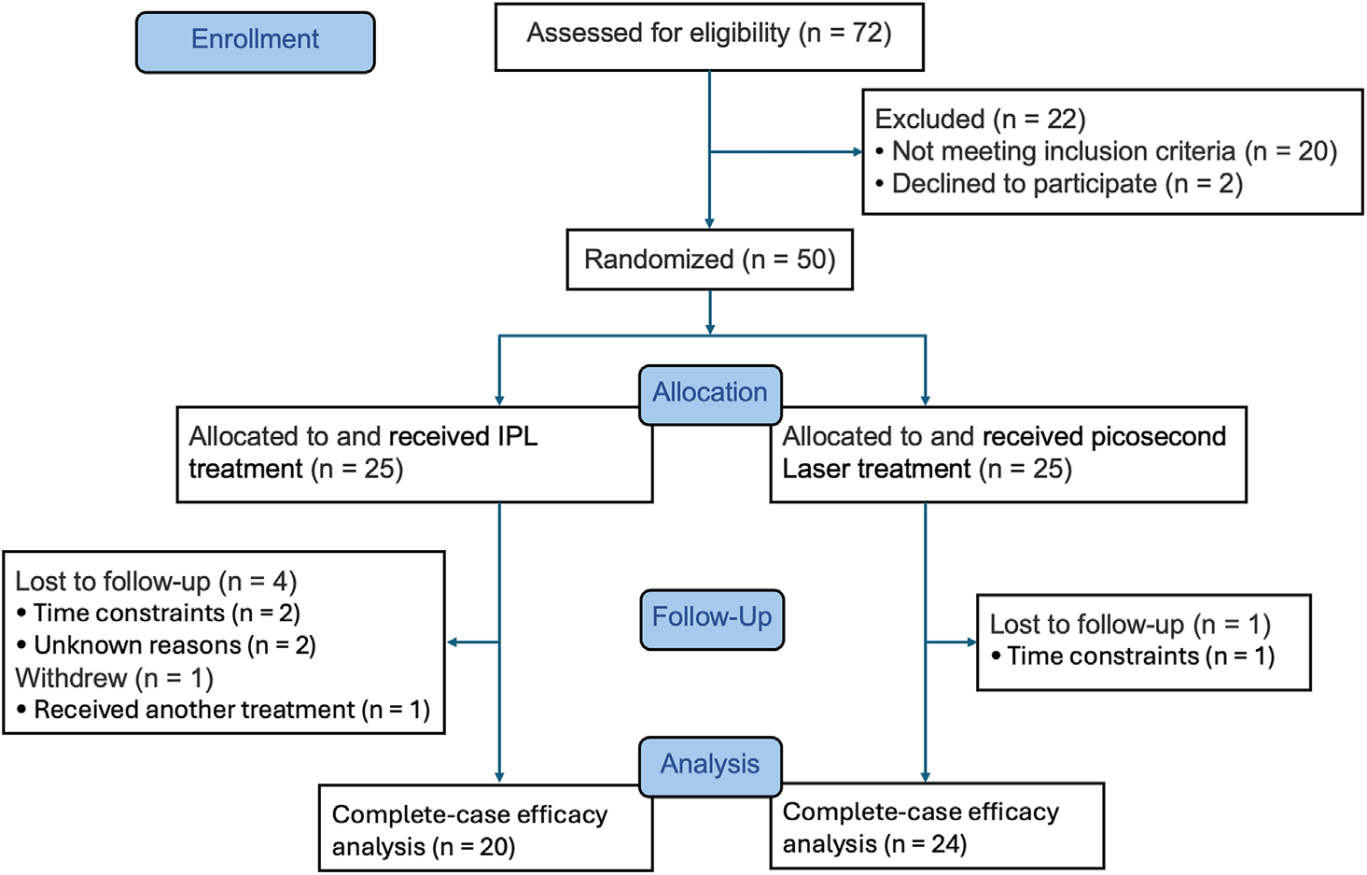
CONSORT flow diagram of participant disposition. IPL, intense pulsed light.

**TABLE 1:** Baseline demographic and clinical characteristics of all randomized participants.

Characteristic	IPL (*n* = 25)	Picosecond laser (*n* = 25)	*χ* ^2^/*Z*	*p*
Gender, *n* (%)				
Female	22 (88.0)	24 (96.0)		0.609^a^
Male	3 (12.0)	1 (4.0)		
Age (years)				
Median (IQR)	26 (24.5–35)	27 (25–29)	0.020	0.984^b^
Min.– Max.	22–60	23–43		
Fitzpatrick skin type, *n* (%)				
II	1 (4.0)	1 (4.0)	1.048	0.790^c^
III	11 (44.0)	10 (40.0)		
IV	13 (52.0)	13 (52.0)		
V	0 (0)	1 (4.0)		
Grading, *n* (%)				
1	17 (68.0)	18 (72.0)	0.105	0.949^c^
2	7 (28.0)	6 (24.0)		
3	1 (4.0)	1 (4.0)		
Family history, *n* (%)				
Yes	10 (40.0)	9 (36.0)	0.085	0.771^c^
No	15 (60.0)	16 (64.0)		
Disease duration (years)				
Median (IQR)	12 (9–18.5)	20 (10–20)	0.780	0.436^b^
Min.– Max.	3–30	3–25		

Abbreviations: IPL, intense pulsed light; IQR, interquartile range.

^a^ Fisher's exact test.

^b^ Wilcoxon rank‐sum test.

^c^ Chi‐square test.

The median age was 26 (IQR, 24.5–35) years in the IPL group and 27 (IQR, 25–29) years in the picosecond laser group. Fitzpatrick skin types ranged from II to V across the study population, with more than 90% of patients in each group classified as Type III or IV. According to the Altalhab classification [4], most patients had Grade 1 lip hyperpigmentation (IPL, 68.0%; picosecond laser, 72.0%), and representative clinical images of each grade are shown in Figure 2. A family history of similar pigmentation was reported in 10 (40.0%) patients in the IPL group and 9 (36.0%) patients in the picosecond laser group. The median disease duration was 12 (IQR, 9–18.5) years in the IPL group and 20 (IQR, 10–20) years in the picosecond laser group. No significant differences were observed in baseline demographic or clinical characteristics between the two groups (all *p* > 0.05).

**FIGURE 2:**
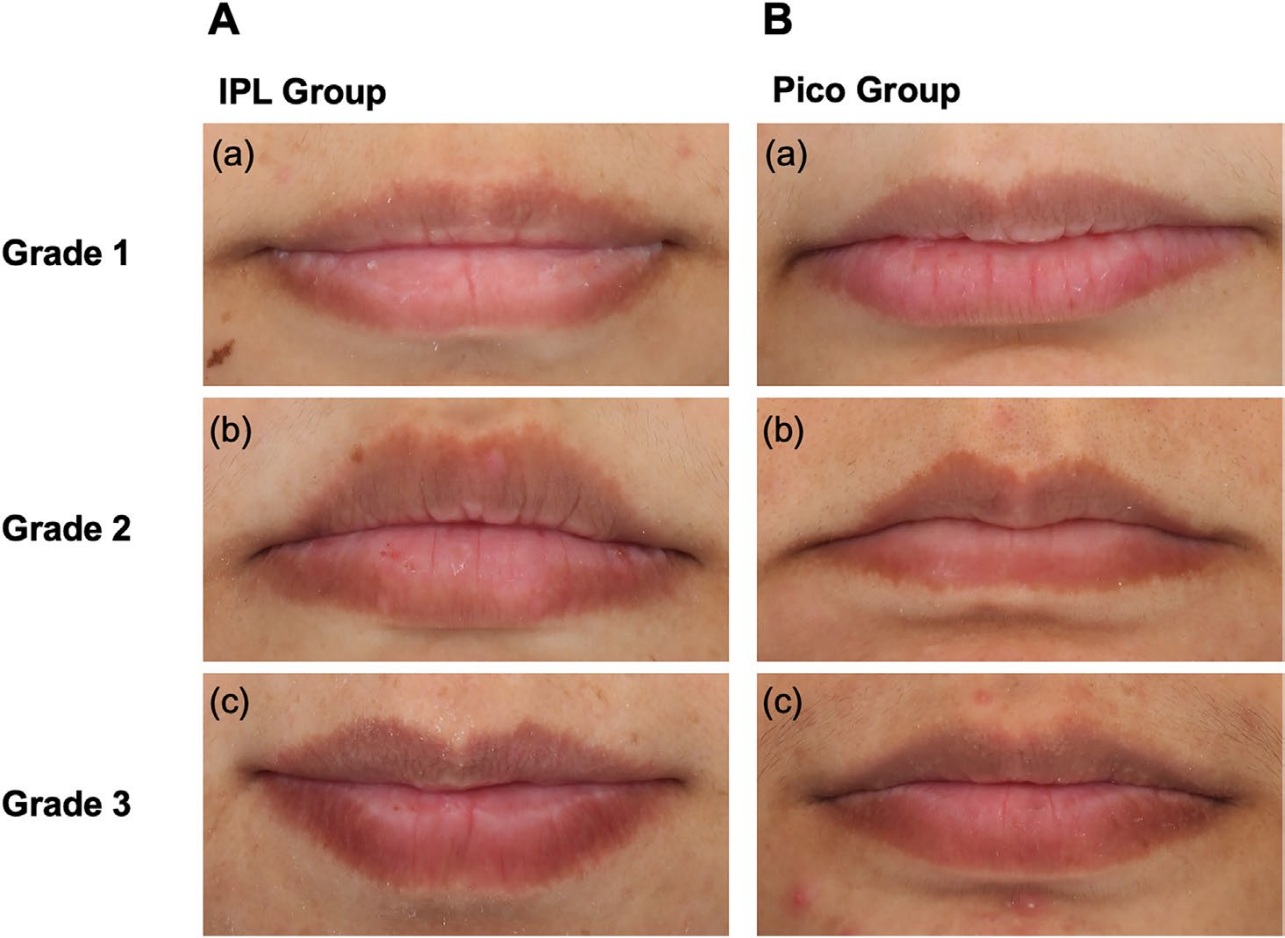
Classification of lip hyperpigmentation and representative cases in the IPL (A) and picosecond laser (B) groups. Lip hyperpigmentation was classified into three grades according to Altalhab et al. [4]: (a) Grade 1, diffuse involvement of the upper lip and the border of the lower lip; (b) Grade 2, involvement of the upper lip and most of the lower lip with sparing of the central portion; and (c) Grade 3, near‐complete involvement of both lips. IPL, intense pulsed light; Pico, picosecond laser.

### 3.2 Efficacy

Representative clinical images at baseline and Week 12 are shown in Figure 3. Inter‐rater reliability for PGA scores yielded an ICC of 0.799 (95% CI, 0.634–0.890). Among completers, the median PGA score at Week 12 was 1.5 (IQR, 1–2.375) in the IPL group and 2.5 (IQR, 2–3) in the picosecond laser group (Figure 4A). PGA scores were significantly higher in the picosecond laser group (*p* = 0.007), with a Hodges–Lehmann estimate of 0.5 PGA points (95% CI, 0–1.0). A PGA score ≥ 2 was achieved in 9 (45.0%) patients in the IPL group and 19 (79.2%) patients in the picosecond laser group (*p* = 0.029; risk difference, 34.2% [95% CI, 5.6–56.6]; risk ratio, 1.76 [95% CI, 1.04–2.98]). In sensitivity analyses, point estimates for PGA scores and PGA ≥ 2 response rates consistently favored the picosecond laser. However, between‐group differences were no longer statistically significant under some assumptions about missing outcomes (Tables S1 and S2).

**FIGURE 3:**
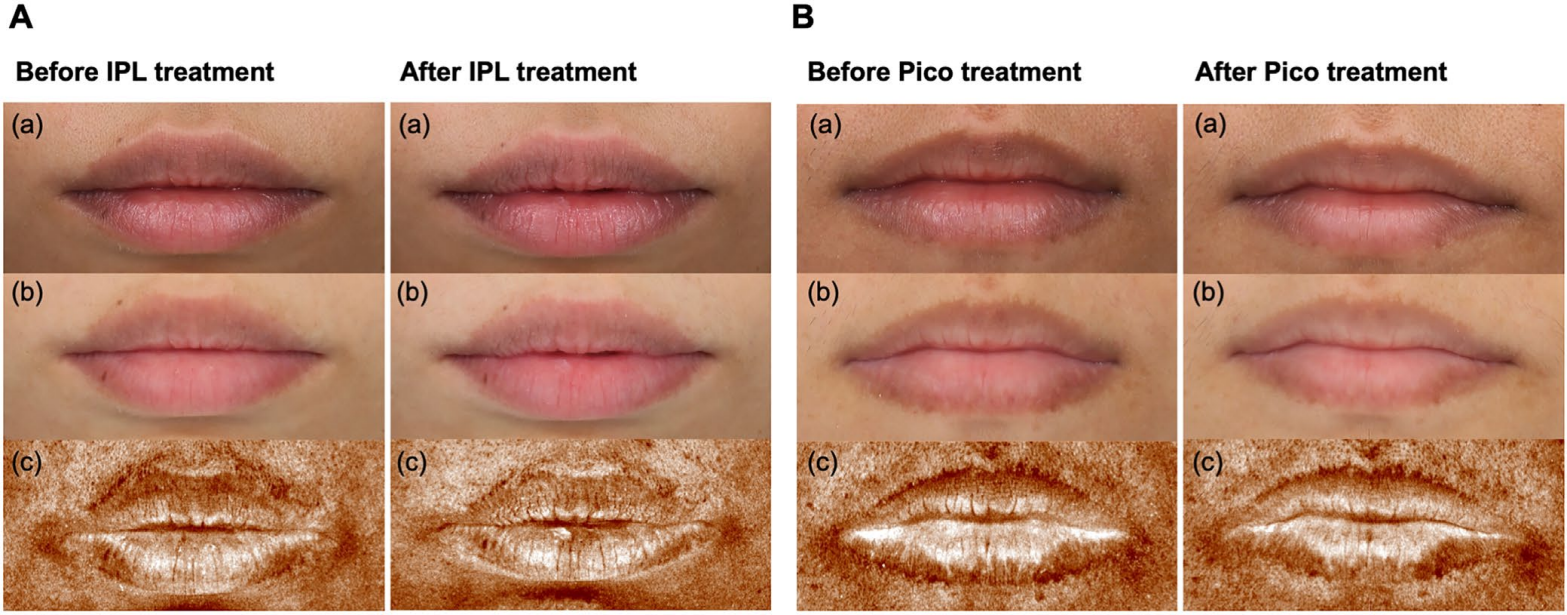
Representative clinical images of lip hyperpigmentation at baseline and Week 12 in the IPL group (A) and the picosecond laser group (B). (a) Standard white light; (b) cross‐polarized; (c) brown spot analysis. IPL, intense pulsed light; Pico, picosecond laser.

**FIGURE 4:**
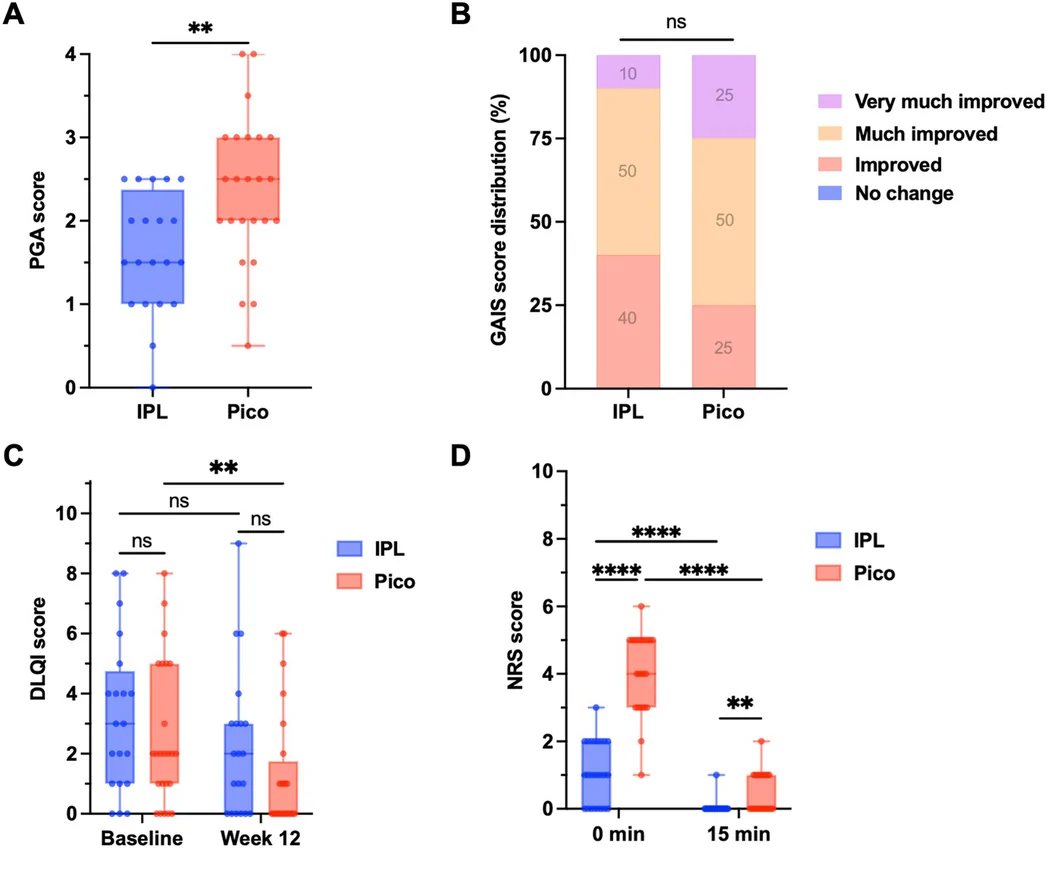
PGA scores and patient‐reported outcomes in the IPL and picosecond laser groups. (A) PGA scores at Week 12; (B) distribution of GAIS scores at Week 12; (C) DLQI scores at baseline and Week 12; (D) pain NRS scores immediately after the first treatment session (0 min) and after 15 min of post‐treatment cooling (15 min). Panels A–C include study completers (IPL, *n* = 20; picosecond laser, *n* = 24); panel D includes all randomized participants (*n* = 25 per group). In panels A, C, and D, boxes represent interquartile ranges, horizontal lines indicate medians, whiskers indicate minimum and maximum values, and dots represent individual participants. ns, not significant; **p < 0.01; ****p < 0.0001. DLQI, Dermatology Life Quality Index; GAIS, Global Aesthetic Improvement Scale; IPL, intense pulsed light; min, minutes; NRS, Numerical Rating Scale; PGA, Physician's Global Assessment; Pico, picosecond laser.

Quantitative color parameters of the upper and lower lips are summarized in Table 2. In the upper lip, L* values increased significantly from baseline to Week 12 in both the IPL group (*p* = 0.002) and the picosecond laser group (*p* = 0.001), whereas b* values decreased significantly only in the picosecond laser group (*p* = 0.038). No significant changes in a* values were observed in either group. In the lower lip, the picosecond laser group showed a significant increase in L* values (*p* = 0.010) and a decrease in b* values (*p* = 0.008), whereas no significant changes were observed in the IPL group. Percentage changes from baseline in L* and b* were greater in the picosecond laser group than in the IPL group for both the upper and lower lips. However, between‐group differences were not statistically significant (all *p* > 0.05).

**TABLE 2:** Lip color parameters before and after treatment in the IPL and picosecond laser groups.

Color parameters	IPL (*n* = 20)			*p* ^1^	Picosecond laser (*n* = 24)			*p* ^2^	Between‐group difference (95% CI)	*p* ^3^
	Baseline	Week 12	Change (%)		Baseline	Week 12	Change (%)			
Upper lip, Mean (SD)										
L*	45.43 (2.36)	46.36 (2.16)	2.10 (2.71)	0.002	44.74 (4.07)	46.61 (4.01)	4.40 (5.96)	0.001	2.30 (−0.47 to 5.06)	0.100
a*	26.96 (1.87)	26.75 (1.91)	−0.65 (5.45)	0.525	25.89 (2.18)	25.48 (3.62)	−1.75 (9.10)	0.418	−1.10 (−5.60 to 3.39)	0.622
b*	17.84 (1.86)	17.75 (1.78)	−0.25 (6.47)	0.746	17.51 (1.78)	16.79 (1.88)	−3.75 (9.15)	0.038	−3.49 (−8.26 to 1.28)	0.147
Lower lip, Mean (SD)										
L*	44.61 (3.49)	45.21 (3.66)	1.43 (5.09)	0.242	43.08 (4.76)	44.40 (4.55)	3.27 (5.48)	0.010	1.84 (−1.38 to 5.06)	0.255
a*	33.84 (2.85)	33.59 (2.74)	−0.49 (7.09)	0.649	32.99 (3.28)	33.00 (3.73)	0.28 (8.82)	0.987	0.77 (−4.07 to 5.61)	0.751
b*	18.57 (2.31)	18.07 (3.01)	−2.76 (12.32)	0.309	18.57 (1.51)	17.55 (2.21)	−5.48 (9.61)	0.008	−2.73 (−9.59 to 4.14)	0.426

*Note:* L*, lightness; a*, red–green axis; b*, yellow–blue axis. *p* ^1^ and *p* ^2^ indicate within‐group comparisons between baseline and Week 12 in the IPL and picosecond laser groups, respectively; *p* ^3^ indicates between‐group comparisons of percentage change. Mean differences were calculated as picosecond laser minus IPL. *p* ^1^ and *p* ^2^ were analyzed using paired *t*‐tests, and *p* ^3^ was analyzed using Welch's *t*‐test.

Abbreviations: CI, confidence interval; IPL, intense pulsed light; SD, standard deviation.

### 3.3 Patient‐Reported Outcomes

At Week 12, 60.0% of patients in the IPL group and 75.0% in the picosecond laser group reported much improved or very much improved on the GAIS (Figure 4B). No patients reported worse or no change. GAIS score distribution did not differ significantly between groups (*p* = 0.372).

The median DLQI score decreased from 3 (IQR, 1–4.75) at baseline to 2 (IQR, 0–3) at Week 12 in the IPL group, and from 2 (IQR, 1–5) to 0 (IQR, 0–1.75) in the picosecond laser group (Figure 4C). The reduction was significant in the picosecond laser group (*p* = 0.008), but not in the IPL group (*p* = 0.154). No significant between‐group differences were observed in DLQI scores at baseline (*p* = 0.384) or Week 12 (*p* = 0.088; Hodges–Lehmann estimate, −1.0 DLQI points; 95% CI, −2.0 to 0.0).

Immediately after the first treatment session, the median pain NRS score was higher in the picosecond laser group than in the IPL group (4 [IQR, 3–5] vs. 1 [IQR, 0–2]; *p* < 0.0001; Hodges–Lehmann estimate, 3 NRS points; 95% CI, 3 to 4). After 15 min of post‐treatment cooling, the between‐group difference remained significant (0 [IQR, 0–1] vs. 0 [IQR, 0–0]; *p* < 0.01; Hodges–Lehmann estimate, 0 NRS points; 95% CI, 0 to 1) (Figure 4D). Pain NRS scores decreased significantly between the two assessments in both groups (both *p* < 0.0001).

### 3.4 Safety

Two patients in the picosecond laser group developed scaling 3 days after treatment, which resolved within 1 week with lip balm. No adverse events were reported in the IPL group. No other adverse reactions, including infection, scarring, hyperpigmentation, or hypopigmentation, were observed during follow‐up.

## 4 Discussion

In this randomized trial, both treatments improved lip pigmentation within 12 weeks and showed favorable overall safety. At Week 12, a higher proportion of patients achieved at least moderate improvement in the 755‐nm picosecond laser group than in the IPL group (79.2% vs. 45.0%). Objective colorimetric analysis showed increased lip lightness and reduced pigmentation following both treatments. All completers reported improvement on the GAIS, and DLQI scores decreased, indicating favorable patient‐perceived aesthetic and quality‐of‐life outcomes. However, the picosecond laser was associated with greater immediate pain after the first treatment session, whereas pain was generally mild in the IPL group, although post‐treatment cooling markedly reduced pain in both groups. Two patients treated with the picosecond laser developed transient scaling, which resolved with lip care, and no treatment‐related adverse events occurred with IPL. These findings suggest that the 755‐nm picosecond laser may provide greater physician‐assessed improvement, whereas IPL may offer better treatment tolerability. However, the efficacy comparison should be interpreted cautiously because statistical significance depended on assumptions about missing outcomes.

Previous studies on the treatment of lip hyperpigmentation are limited and have mainly focused on Q‐switched Nd:YAG 532‐nm lasers (QS 532‐nm); most are small retrospective studies, prospective pilot studies, or case reports [2, 4, 21, 22]. Reported adverse events include erythema, crusting, recurrence of herpes labialis, worsening pigmentation, and pigmentary mottling; recurrence has also been described in some cases [2, 4, 21, 22]. Mechanistically, the 532‐nm wavelength is highly absorbed by epidermal melanin and is therefore more likely to cause epidermal injury, increasing the risk of post‐inflammatory hyperpigmentation (PIH) [23]. This risk is particularly relevant in Asian skin, as higher epidermal melanin content may increase susceptibility to PIH following laser treatment [24].

In the present study, the 755‐nm picosecond laser showed a favorable balance between efficacy and safety. A previous pilot study from Thailand included 20 patients with physiological lip hyperpigmentation [25]. After five sessions of 755‐nm picosecond laser treatment, 52.6% of patients achieved moderate improvement, the mean melanin index decreased significantly, and only one case of transient edema was reported, providing preliminary evidence for the efficacy and safety of this modality [25]. Our randomized controlled study further supports these findings. Compared with conventional nanosecond lasers, picosecond lasers have a shorter pulse duration and higher peak power density, producing a stronger photomechanical effect while reducing thermal diffusion to surrounding tissues [16]. This allows more efficient pigment clearance with less non‐target thermal injury. This may help explain the greater physician‐assessed improvement observed among completers while maintaining an acceptable safety profile.

Unlike single‐wavelength lasers, IPL is a broad‐spectrum pulsed light source that emits light at wavelengths of 400–1400 nm [26]. Its output spectrum can be adjusted with cut‐off filters to target melanin or hemoglobin through selective photothermolysis [26]. IPL has been widely used for pigmentary disorders, vascular lesions, and skin rejuvenation, and is generally associated with fewer adverse effects and less pain [26]. The lip has a thinner stratum corneum, a weaker barrier, and relatively lower melanin content, and is more sensitive to irritation and pain [11]. Therefore, compared with more concentrated laser energy, IPL may be better tolerated at this site. Clinical evidence for IPL in lip hyperpigmentation remains scarce, and our study helps fill this gap.

This study also used objective colorimetric parameters to quantify treatment response. Previous studies have mainly relied on subjective assessment. Some used devices such as the Mexameter MX16 or Antera 3D CS to objectively measure the melanin index, but the former is contact‐based and may be affected by probe placement and applied pressure [14, 25]. In contrast, our study extracted CIELAB parameters from standardized polarized VISIA images. This approach may help reduce operator‐dependent bias. In addition, colorimetric analyses were performed separately for the upper and lower lips because baseline lip color differs between these regions and lower‐lip hyperpigmentation was mainly distributed along the lateral vermilion border rather than the central vermilion [4, 27]. After both treatments, the main objective changes were an increase in L* and a decrease in b*, whereas changes in a* were small. Between‐group differences were not statistically significant.

This study has several limitations. First, no formal sample size calculation was performed, and the sample size was small; therefore, the findings should be interpreted as exploratory. Second, this was a single‐center study conducted in a Chinese population, which limits the generalizability of the findings. Third, the follow‐up period of 4 weeks after the final treatment session was relatively short and insufficient to fully assess long‐term treatment durability, delayed hyperpigmentation, or recurrence risk. Fourth, the complete‐case efficacy analysis and greater attrition in the IPL group may have introduced bias that was not fully accounted for in the sensitivity analyses. Further multicenter studies with larger sample sizes and longer follow‐up are needed to define the optimal treatment strategy for diffuse lip hyperpigmentation.

## 5 Conclusion

Both the 755‐nm picosecond laser and IPL effectively improved diffuse lip hyperpigmentation, with favorable overall safety and tolerability. Among completers, the 755‐nm picosecond laser showed greater physician‐assessed improvement in pigmentation, whereas IPL was better tolerated. However, the efficacy comparison should be interpreted cautiously because of unequal attrition and sensitivity to missing‐outcome assumptions. Objective colorimetric measurements showed no statistically significant between‐group differences. Further multicenter studies with larger sample sizes and longer follow‐up are needed to validate these findings and define the optimal treatment strategy.

## Author Contributions

Hongfei Ouyang and Ruixing Yu designed the research study. Hongfei Ouyang, Jiahui Han, Lili Chen, and Xuejiao Song, and Ruixing Yu were involved in data collection and data analysis. Hongfei Ouyang drafted the article. Ruixing Yu and Yong Cui were involved in the key revision of the article. All authors were involved in the final approval of the version to be published.

## Funding

This study was supported by the National High Level Hospital Clinical Research Funding (Grant No. 2025‐NHLHCRF‐YXHZ‐MS‐03), the Key Project of the Capacity Building and Continuing Education Center (Party School of the National Health Commission), National Health Commission of the People's Republic of China (Grant No. GWJJMB202510025047), and the Horizontal Project of China‐Japan Friendship Hospital (Grant No. 2025‐HX‐149).

## Ethics Statement

The study protocol was approved by the Ethics Committee of China–Japan Friendship Hospital, Beijing, China (2025‐KY‐294‐1).

## Consent

Written informed consent was obtained from all participants prior to enrollment, including the consent for publication of the clinical images.

## Conflicts of Interest

The authors declare no conflicts of interest.

## Supporting information


**Figure S1:** Standardized placement of regions of interest for objective colorimetric analysis. Six 50 × 50‐pixel regions of interest (ROIs) were used: four at standardized quintile positions on the upper lip and two on the lateral lower lip. The central lower lip was excluded because pigmentation was predominantly distributed along the lateral vermilion border. Mean values were calculated separately for the upper and lower lips.
**Table S1:** Sensitivity analyses for missing Week‐12 PGA scores.
**Table S2:** Sensitivity analyses of PGA ≥ 2 response rates.

## Data Availability

The data are available on reasonable request from the corresponding author. The data are not publicly available due to privacy or ethical restrictions.
