# Supplementary material for: 755‐nm Picosecond Laser Versus Intense Pulsed Light for Diffuse Lip Hyperpigmentation: A Prospective, Randomized, Controlled, Evaluator‐Blinded Trial
Source: J Cosmet Dermatol. 2026 Nov 13;25(11):e71295. doi: 10.1111/jocd.71295 (PMC13768460; doi:10.1111/jocd.71295)
Supplement: Supplementary file 1 [file JOCD-25-e71295-s001.docx]

**Supplementary Appendix**

**Supplement to:**

755-nm Picosecond Laser versus Intense Pulsed Light for Diffuse Lip Hyperpigmentation: A Prospective, Randomized, Controlled, Evaluator-Blinded Trial

**Supplementary Figure S1.** Standardized placement of regions of interest for objective colorimetric analysis. Six 50 × 50-pixel regions of interest (ROIs) were used: four at standardized quintile positions on the upper lip and two on the lateral lower lip. The central lower lip was excluded because pigmentation was predominantly distributed along the lateral vermilion border. Mean values were calculated separately for the upper and lower lips.

**Supplementary Table S1. Sensitivity analyses for missing week-12 PGA scores**

**Panel A. *p* values for between-group comparisons**

| **Assumed PGA score for picosecond laser** | **Assumed PGA score for IPL** | | | | | | | | |
| --- | --- | --- | --- | --- | --- | --- | --- | --- | --- |
|  | **0** | **0.5** | **1** | **1.5** | **2** | **2.5** | **3** | **3.5** | **4** |
| **0** | 0.0011 | 0.0016 | 0.0026 | 0.0051 | 0.0167 | 0.0676 | 0.1874 | 0.3080 | 0.3827 |
| **0.5** | 0.0008 | 0.0012 | 0.0024 | 0.0048 | 0.0158 | 0.0643 | 0.1806 | 0.2985 | 0.3720 |
| **1** | 0.0007 | 0.0008 | 0.0017 | 0.0041 | 0.0135 | 0.0570 | 0.1668 | 0.2762 | 0.3455 |
| **1.5** | 0.0005 | 0.0006 | 0.0010 | 0.0025 | 0.0103 | 0.0462 | 0.1396 | 0.2388 | 0.3026 |
| **2** | 0.0003 | 0.0004 | 0.0007 | 0.0015 | 0.0066 | 0.0370 | 0.1164 | 0.2044 | 0.2630 |
| **2.5** | 0.0002 | 0.0003 | 0.0005 | 0.0011 | 0.0042 | 0.0256 | 0.0972 | 0.1750 | 0.2266 |
| **3** | 0.0002 | 0.0002 | 0.0004 | 0.0009 | 0.0036 | 0.0198 | 0.0787 | 0.1600 | 0.2087 |
| **3.5** | 0.0002 | 0.0002 | 0.0004 | 0.0009 | 0.0036 | 0.0198 | 0.0709 | 0.1459 | 0.2090 |
| **4** | 0.0002 | 0.0002 | 0.0004 | 0.0009 | 0.0036 | 0.0199 | 0.0708 | 0.1328 | 0.1915 |

**Panel B. Hodges–Lehmann estimates of between-group differences (PGA points)**

| **Assumed PGA score for picosecond laser** | **Assumed PGA score for IPL** | | | | | | | | |
| --- | --- | --- | --- | --- | --- | --- | --- | --- | --- |
|  | **0** | **0.5** | **1** | **1.5** | **2** | **2.5** | **3** | **3.5** | **4** |
| **0** | 1.0 | 1.0 | 1.0 | 0.5 | 0.5 | 0.5 | 0.5 | 0.5 | 0.5 |
| **0.5** | 1.0 | 1.0 | 1.0 | 0.5 | 0.5 | 0.5 | 0.5 | 0.5 | 0.5 |
| **1** | 1.0 | 1.0 | 1.0 | 0.5 | 0.5 | 0.5 | 0.5 | 0.5 | 0.5 |
| **1.5** | 1.0 | 1.0 | 1.0 | 0.5 | 0.5 | 0.5 | 0.5 | 0.5 | 0.5 |
| **2** | 1.0 | 1.0 | 1.0 | 0.5 | 0.5 | 0.5 | 0.5 | 0.5 | 0.5 |
| **2.5** | 1.0 | 1.0 | 1.0 | 1.0 | 0.5 | 0.5 | 0.5 | 0.5 | 0.5 |
| **3** | 1.0 | 1.0 | 1.0 | 1.0 | 0.5 | 0.5 | 0.5 | 0.5 | 0.5 |
| **3.5** | 1.0 | 1.0 | 1.0 | 1.0 | 0.5 | 0.5 | 0.5 | 0.5 | 0.5 |
| **4** | 1.0 | 1.0 | 1.0 | 1.0 | 0.5 | 0.5 | 0.5 | 0.5 | 0.5 |

**Notes:** Row values indicate the score assigned to the one missing picosecond laser participant; column values indicate the common score assigned to all five missing IPL participants. All 44 observed scores were retained, yielding 81 scenarios with 25 participants per group. Panel A reports *p* values from exact permutation Mann–Whitney U tests accounting for ties. Panel B reports Hodges–Lehmann estimates (picosecond laser minus IPL); positive values favor the picosecond laser. PGA, Physician’s Global Assessment; IPL, intense pulsed light.

**Supplementary Table S2. Sensitivity analyses of PGA ≥2 response rates**

| **Assumed responders among missing participants, n** | |  | **Total responders, n/N (%)** | | **Risk difference (95% CI)** | ***p*** |
| --- | --- | --- | --- | --- | --- | --- |
| **Picosecond laser** | **IPL** |  | **Picosecond laser** | **IPL** |  |  |
| Observed completers | |  | 19/24 (79.2) | 9/20 (45.0) | 34.2 (5.6 to 56.6) | 0.0285 |
| 0 | 0 |  | 19/25 (76.0) | 9/25 (36.0) | 40.0 (12.5 to 60.1) | 0.0096 |
| 0 | 1 |  | 19/25 (76.0) | 10/25 (40.0) | 36.0 (8.6 to 56.8) | 0.0209 |
| 0 | 2 |  | 19/25 (76.0) | 11/25 (44.0) | 32.0 (4.9 to 53.4) | 0.0421 |
| 0 | 3 |  | 19/25 (76.0) | 12/25 (48.0) | 28.0 (1.2 to 49.9) | 0.0792 |
| 0 | 4 |  | 19/25 (76.0) | 13/25 (52.0) | 24.0 (−2.5 to 46.3) | 0.1398 |
| 0 | 5 |  | 19/25 (76.0) | 14/25 (56.0) | 20.0 (−6.0 to 42.7) | 0.2321 |
| 1 | 0 |  | 20/25 (80.0) | 9/25 (36.0) | 44.0 (16.7 to 63.3) | 0.0037 |
| 1 | 1 |  | 20/25 (80.0) | 10/25 (40.0) | 40.0 (12.9 to 60.0) | 0.0086 |
| 1 | 2 |  | 20/25 (80.0) | 11/25 (44.0) | 36.0 (9.1 to 56.6) | 0.0186 |
| 1 | 3 |  | 20/25 (80.0) | 12/25 (48.0) | 32.0 (5.4 to 53.1) | 0.0378 |
| 1 | 4 |  | 20/25 (80.0) | 13/25 (52.0) | 28.0 (1.8 to 49.6) | 0.0718 |
| 1 | 5 |  | 20/25 (80.0) | 14/25 (56.0) | 24.0 (−1.8 to 46.0) | 0.1284 |

**Notes:** Response was defined as a mean PGA score ≥2. The first row presents observed completers. The remaining rows show 12 combinations of assumed responder counts among the missing participants: 0–1 for picosecond laser and 0–5 for IPL. Observed outcomes were retained, with 25 participants per group in each scenario. Risk differences are picosecond laser minus IPL, in percentage points. Fisher’s exact tests and Newcombe–Wilson 95% CIs were used. PGA, Physician’s Global Assessment; IPL, intense pulsed light; CI, confidence interval.
